# An in vitro study evaluating the effect of ferrule design on the fracture resistance of endodontically treated mandibular premolars after simulated crown lengthening or forced eruption methods

**DOI:** 10.1186/s12903-018-0549-8

**Published:** 2018-05-10

**Authors:** Qingfei Meng, Qian Ma, Tianda Wang, Yaming Chen

**Affiliations:** 1 Department of Stomatology, Xuzhou Central Hospital, Xuzhou, 221009 Jiangsu Province China; 2 College of Stomatology, Nanjing Medical University, 136 Hanzhong Road, Nanjing, 210029 Jiangsu Province China

**Keywords:** Ferrule, Surgical crown lengthening, Orthodontic forced eruption, Fracture resistance, Residual root

## Abstract

**Background:**

The purpose of this study was to evaluate the effect of ferrule design on the fracture resistance of endodontically treated mandibular first premolars after simulated crown lengthening and orthodontic forced eruption methods restored with a fiber post-and-core system.

**Methods:**

Forty extracted and endodontically treated mandibular first premolars were decoronated to create lingual-to-buccal oblique residual root models, with a 2.0 mm height of the lingual dentine wall coronal to the cemento-enamel junction, and the height of buccal surface at the cemento-enamel junction. The roots were divided randomly into five equal groups. The control group had undergone incomplete ferrule preparation in the cervical root, with 0.0 mm buccal and 2.0 mm lingual ferrule lengths (Group F0). Simulated surgical crown lengthening method provided ferrule preparation of 1.0 mm (Group CL/F1) and 2.0 mm (Group CL/F2) on the buccal surface, with ferrule lengths of 3.0 mm and 4.0 mm on the lingual surface, respectively. Simulated orthodontic forced eruption method provided ferrule preparation of 1.0 mm (Group OE/F1) and 2.0 mm (Group OE/F2) on the buccal surface and ferrule lengths of 3.0 mm and 4.0 mm on the lingual surface, respectively. After restoration with a glass fiber post-and-core system and a cast Co-Cr alloy crown, each specimen was embedded in an acrylic resin block to a height on the root 2.0 mm from the apical surface of the crown margin and loaded to fracture at a 135° angle to its long axis in a universal testing machine. Data were analyzed statistically using two-way ANOVA with Tukey HSD tests and Fisher’s test, with α = 0.05.

**Results:**

Mean fracture loads (kN) for groups F0, CL/F1, CL/F2, OE/F1 and OE/F2 were as follows: 1.01 (S.D. = 0.26), 0.91 (0.29), 0.73 (0.19), 0.96 (0.25) and 0.76 (0.20), respectively. Two-way ANOVA revealed significant differences for the effect of ferrule lengths (*P* = 0.012) but no differences for the effect of cervical treatment methods (*P* = 0.699). The teeth with no buccal ferrule preparation in control group F0 had the highest fracture resistance. In contrast, the mean fracture loads for group CL/F2 with a 2.0-mm buccal and 4.0-mm lingual ferrule created by simulated crown lengthening method were lowest (*P* = 0.036).

**Conclusions:**

Increased apically complete ferrule preparation resulted in decreased fracture resistance of endodontically treated mandibular first premolars, regardless of whether surgical crown lengthening or orthodontic forced eruption methods been used.

## Background

When endodontically treated teeth are restored with a post-and-core system, the prognosis can be affected by the following factors: the remaining amount of residual tooth [1–4], ferrule design [3, 5–8], post material [3, 5, 6], the tooth fracture mode and its severity [9, 10], and so on. According to previous studies [1–4], the amount of residual tooth structure has been considered the most important factor with regard to the fracture resistance of endodontically treated teeth. If adequate supragingival tooth structure could be conserved and greater than a 1.0-mm complete ferrule could be achieved, the fracture resistance would increase significantly and the long-term success of post-and-core restorations could be expected [7, 8].

However, because of severe dental caries, wedge-shaped defects, trauma or other reasons, teeth were broken obliquely in some cases, starting at the crown and extending longitudinally through the pulp chamber to the cervical line or subgingival area, with one or more dentine walls lost and only an incomplete ferrule prepared in the residual cervical root. Mangold et al. had evaluated the fracture resistance of endodontically-treated residual teeth in this particular condition and found that the fracture loads of such obliquely broken teeth decreased proportionally with the loss of dentine walls [11–13]. Moreover, the position of the lost dentine wall would still affect the long-term success of oblique endodontic root restorations [14, 15]. According to previous studies [14, 15], the fracture resistance of anterior upper teeth having a 2.0-mm lingual ferrule (labial dentine wall was lost) was better than those with a 2.0-mm labial ferrule (lingual dentine wall was lost). The lowest fracture resistance was found in residual roots with no ferrule or only a labial ferrule. The long-term prognosis for such residual roots appears to be poor after post-and-core restorations.

The surgical crown lengthening method (SCLM) and orthodontic forced eruption method (OFEM) have been recommended for restoring subgingival roots [16–20]. SCLM reestablishes the dentogingival junction at a more apical level on the root to accommodate the junctional epithelium and the connective tissue attachment in a relatively short time frame [16–18]. In contrast, continued, slow, passive or active OFEM at approximately 2 mm per month allows the periodontal ligament to repair and the alveolar bone to remodel by orthodontic adjustments [19, 20]. Both cervical treatment methods can provide sound tooth structure over the bone crest for the complete ferrule preparation. OFEM has been shown to provide greater fracture resistance and better clinical outcome for post-and-core restored roots than SCLM [4, 8]. However, whether SCLM or OFEM is supportive for residual roots with an oblique fracture is still unclear.

The purpose of this in vitro study was to investigate the effect of ferrule length on the fracture resistance of obliquely-fractured (with buccal tooth structure lost) endodontically treated mandibular premolars, which were treated with simulated SCLM or OFEM to provide a complete ferrule and restored with a prefabricated fiber post and core system. The null hypotheses were that the fracture resistance of endodontically-treated residual roots would not be affected by the ferrule length, nor by the cervical treatment methods (SCLM or OFEM).

## Methods

### Specimen preparation

**Fig. 1:**
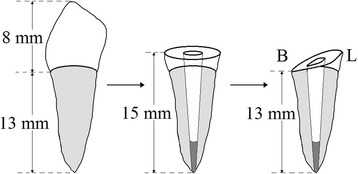
Process of lingual-to-buccal oblique residual root preparation. B: buccal surface of root; L: lingual surface of root

Forty healthy human mandibular first premolars recently-extracted for orthodontic reasons from patients aged 20–30 years who lived in the same locality without water fluoridation were used for this study. Written informed consent was obtained under a protocol approved by the Ethics Committee of Affiliated Stomatology Hospital of Nanjing Medical University. After cleaning and the removal of attached soft tissues, the teeth were examined stereoscopically at 10× magnification to exclude those already cracked before being stored in 0.9% saline solution at 4 °C for no longer than 2 weeks [8]. Diamond disks were used to section the natural crowns transversely, 2 mm occlusal to the buccal cemento-enamel junction (CEJ) with an average root length of 15.0 ± 1.0 mm to simulate endodontically treated roots (Fig. 1). All the roots were assigned randomly into five equal groups (F0, CL/F1, CL/F2, OE/F1 and OE/F2), according to a table of random numbers. The root dimensions including the root lengths, the cross-sectional widths of the canal walls at the mesial, buccal, distal and lingual root face sites and the mesiodistal and buccolingual diameters of the roots were measured to 0.02 mm with a vernier caliper (Vernier Caliper Model 93,218–0654, Harbin Measuring & Cutting Tool Group Co. Ltd., Harbin, PR China). The roots had similar dimensions among the five groups, as shown in Table 1.

**Table 1:** Mean dimensions (mm) of randomly assigned mandibular first premolar roots in each group

Group (*N* = 40)	Root Length^a^	Width of canal wall sites, and roots, at root face^a^					
		Mesial	Buccal	Distal	Lingual	M-D	B-L
F0	15.12 (0.36)	2.19 (0.30)	2.55 (0.15)	2.17 (0.41)	2.36 (0.26)	6.18 (0.97)	8.16 (0.73)
CL/F1	15.16 (0.35)	2.04 (0.41)	2.40 (0.43)	1.92 (0.19)	2.43 (0.20)	5.66 (0.58)	7.88 (0.63)
CL/F2	15.18 (0.27)	2.47 (0.54)	2.63 (0.37)	2.12 (0.33)	2.60 (0.34)	6.40 (0.77)	8.25 (0.52)
OE/F1	15.12 (0.26)	2.06 (0.37)	2.47 (0.27)	2.00 (0.42)	2.52 (0.38)	5.89 (0.96)	8.07 (0.70)
OE/F2	15.19 (0.42)	2.18 (0.30)	2.65 (0.30)	2.11 (0.33)	2.66 (0.36)	6.26 (0.60)	8.13 (0.35)
1-way ANOVA	F = 0.083 *P* = 0.987	F = 1.502 *P* = 0.223	F = 0.823 *P* = 0.519	F = 0.691 *P* = 0.603	F = 1.2 *P* = 0.328	F = 1.140 *P* = 0.354	F = 0.412 *P* = 0.799

Group F0: glass fiber post-core with 0.0 mm buccal and 2.0 mm lingual ferrule lengths, as control; Group CL/F1: glass fiber post-core with simulated crown lengthening and 1.0 mm buccal and 3.0 mm lingual ferrule lengths; Group CL/F2: glass fiber post-core with simulated crown lengthening and 2.0 mm buccal and 4.0 mm lingual ferrule lengths; Group OE/F1: glass fiber post-core with simulated orthodontic forced tooth eruption and 1.0 mm buccal and 3.0 mm lingual ferrule lengths; Group OE/F2: glass fiber post-core with simulated orthodontic forced tooth eruption and 2.0 mm buccal and 4.0 mm lingual ferrule lengths

^a^Mean (Standard Deviation); M-D: mesiodistal root width; B-L: buccolingual root width

The root canals were prepared with hand files (K-files, Dentsply-Maillefer, Ballaigues, Switzerland) and size 4 Gates-Glidden drills (Dentsply-Maillefer) for the purpose of standardized canal forms [8], rinsed with 2.5% sodium hypochlorite solution, dried with paper points, and then applied with a thin layer of sealer (AH Plus, Dentsply Detrey, Konstanz, Germany). The cold laterally-condensed gutta percha points (Dentsply International Inc., York, PA, USA) were placed to obturate the canals. After endodontic treatment, all the residual roots were cut as lingual-to-buccal obliquely-broken root models, starting at the middle point of the lingual section and extending longitudinally to the middle point of the buccal CEJ, with the height of buccal dentine wall 2.0 mm lower than that of the lingual position in the section area (Fig. 1). Each root was restored with prefabricated glass fiber post-and-core system. Post space was prepared to 10 mm deep for No. 2 glass fiber post (#2, R.T.D., France), using matching drills with a slow speed contra angle handpiece, according to the manufacturer’s instructions. The prepared root wall was first etched with 32% phosphoric acid gel (UNI-ETCH, BISCO, Inc., Schaumburg, IL, USA) for 15 s and then rinsed thoroughly with an air-water spray and dried lightly with paper points. A resin-based adhesive (ONE-STEP PLUS, BISCO, Inc) was applied twice as a thin layer over the walls of the root wall and once over the surfaces of a prefabricated glass fiber post. After thinning lightly with dry oil-free air, the adhesive was light-cured for 10 s at 600 mW/cm^2^ (Variable Intensity Polymerizer Junior, BISCO, Inc). The post-hole was filled completely by injecting resin luting cement (DUAL-LINK luting cement, BISCO, Inc) into which the fiber post was inserted. This was followed by light-curing for 40 s from a coronal direction. A resin composited core (Light-Core™, Bisco, Inc) was built up around the post and light-cured again for 40 s.

**Fig. 2:**
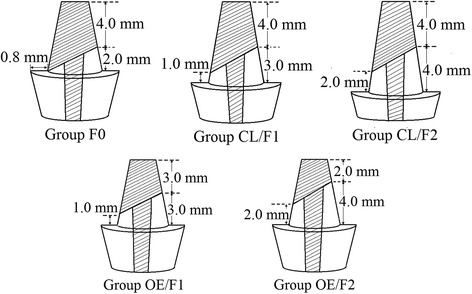
Preparation designs. F0: with 0.0 mm buccal and 2.0 mm lingual ferrule lengths, as control; CL/F1: with simulated crown lengthening and 1.0 mm buccal and 3.0 mm lingual ferrule lengths; CL/F2: with simulated crown lengthening and 2.0 mm buccal and 4.0 mm lingual ferrule lengths; OE/F1: with simulated orthodontic forced tooth eruption and 1.0 mm buccal and 3.0 mm lingual ferrule lengths; OE/F2: with simulated orthodontic forced tooth eruption and 2.0 mm buccal and 4.0 mm lingual ferrule lengths

Cores, 6.0–8.0 mm high with a 6° convergence angle, were prepared using a milling machine (F3/Egro, Degussa AG, Dusseldorf, Germany) with flat-ended tapered carbide burs, leaving a 0.8 mm wide encircling shoulder in dentine. The incomplete ferrule was designed with no ferrule on the buccal surface and 2.0 mm-high ferrule on the lingual surface in the control group (Group F0), which had a 6.0 mm high core. Simulated SCLM resulted in nonuniform circumferential ferrule preparation of 1.0 mm length (Group CL/F1) and of 2.0 mm length (Group CL/F2) in the buccal surface, with the ferrule prepared 3.0 and 4.0 mm length respectively in the lingual surface of the oblique residual roots, which increased the height of the core to 7.0 mm and 8.0 mm, respectively. Simulated OFEM resulted in complete ferrule preparation of 1.0 mm (Group OE/F1) and 2.0 mm (Group OE/F2) on the buccal surface, with the ferrule length at 3.0 and 4.0 mm, respectively, on the lingual surface, while maintaining the 6.0 mm height of the core (as shown in Fig. 2).

After 24 h in the isotonic saline storage medium, a standardized Cobalt-Chromium (Co-Cr) alloy (BEGO Bremer Goldschlägerei Wilh. Herbst GmbH & Co. KG, Germany) crown fabricated in the dental laboratory for each of the prepared teeth was cemented with glass-ionomer cement (Glasionomer, Shofu Inc., Kyoto, Japan). The teeth were kept in the storage medium at all time except during experimental testing.

**Fig. 3:**
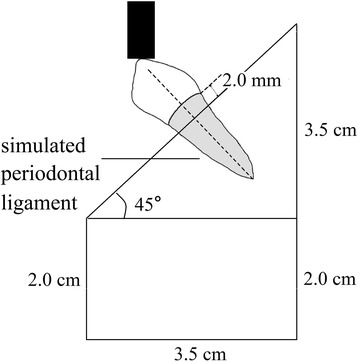
Diagrammatic representation of specimen embedding and loading

Each root was coated with a 0.1–0.2 mm thin vinyl polysiloxane silicone layer (modulus of elasticity 0.3 MPa) (Aquasil, Dentsply International Inc) to simulate the periodontal ligament before being embedded, from 2.0 mm apical to the crown preparation margins, in a block of self-cured acrylic resin (Shanghai Dental Materials Manufacture Co., Shanghai, PR China) (Fig. 3).

### Fatigue resistance testing

A 1,200,000-times dynamic load of 0–50 N, which simulated the oral masticating condition, was applied to the buccal cusp of the Co-Cr alloy crown, at an angle of 135° from the long axis of the root, using a cylindrical Ni-Cr alloy rod (4.0 cm long × 1.2 cm diameter) in a universal load-testing system (MTS810, MTS Systems Co., USA), with the frequency of 1.6 Hz [3]. The failed specimens were recorded and the failure site pattern noted.

### Fracture resistance testing

A unidirectional static load was then applied to the buccal cusp of the Co-Cr alloy crown of the specimen that had passed fatigue testing without failure, with the same load-testing machine (MTS810, MTS Systems Co., USA) and the same load angle at a cross-speed of 0.5 mm/minute (Fig. 3). The force (kilonewton, kN) for initial root fracture was recorded and the failure pattern noted. The fracture modes were divided in repairable (less severe fractures located at or above the cervical third of the roots and potentially repairable) and irrepairable (catastrophic fractures, such as vertical or oblique root fracture located below the cervical third of the roots), according to the root fracture sites.

### Statistical analysis

Statistical analysis was performed using SPSS 21.0 for Windows (SPSS Inc., Chicago, IL, USA). Two-way ANOVA with the Tukey HSD test and Fisher’s exact test were used to detect any significant differences between the groups. The probability level for statistical significance was set at α = 0.05.

## Results

No specimen failed during the fatigue resistance testing. The mean forces required to fracture the restored teeth and the specimen fracture patterns in each group are shown in Table 2. For fracture resistance, 2-way ANOVA revealed a statistically significant difference in the effect of ferrule length (F = 4.955, *P* = 0.012) but no significant differences in the effect of cervical treatment methods (F = 0.152, *P* = 0.699) or interactions between the two sources of variation (F = 0.043, *P* = 0.958) were discerned (Table 3).

**Table 2:** Mean force (kN) required to fracture the tooth roots and the root fracture sites, in each group

Groups	Fatigue testing	Fracture strength (kN)^a^	Root fracture sites^b^	
			At or above cervical 1/3	Below cervical 1/3
F0	0/8	1.01(0.26)	7	1
CL/F1	0/8	0.91 (0.29)	6	2
CL/F2	0/8	0.73 (0.19)	8	0
OE/F1	0/8	0.96 (0.25)	6	2
OE/F2	0/8	0.76 (0.20)	7	1

Group codes are defined in Table 1

^a^Mean (Standard Deviation)

^b^Failed roots were classified repairable (the fracture sites located at or above cervical one-third), and irrepairable (the fracture sites below cervical one-third)

**Table 3:** ANOVA table representing effective decomposition of main variables and their interaction

Source	Sum of Squares	df	Mean Square	F	*P*
Ferrule	0.580	2	0.290	4.955	0.012^a^
Treatment method	0.009	1	0.009	0.152	0.699
Interaction	0.005	2	0.003	0.043	0.958
Error	2.460	42	0.059		

^a^Statistically significant

For the simulated SCLM and OFEM, no significant differences were found in fracture resistance between groups CL/F1 and CL/F2 and between groups OE/F1 and OE/F2 (*P* > 0.05). Only the fracture loads of group CL/F2 were significantly lower than that of the control group (Group F0) (*P* = 0.036) (Table 4). The control group F0 had the highest fracture resistance, and the fracture loads of the obliquely-broken residual roots decreased along with the increasing ferrule lengths regardless of whether SCLM or OFEM had been used.

**Table 4:** Statistical comparisons between groups using Tukey HSD tests

Buccal Ferrule length	Simulated crown lengthening	Simulated forced eruption
0.0 mm/1.0 mm	*P* = 0.457	*P* = 0.712
0.0 mm/2.0 mm	*P* = 0.036^a^	*P* = 0.052
1.0 mm/2.0 mm	*P* = 0.154	*P* = 0.107

^a^Statistically significant

Almost all the fracture lines were found at or above the cervical one-third of the roots (Table 2). No statistically significant differences in fracture modes were found among the groups by Fisher’s exact test (*P* = 1.00).

## Discussion

### Specimen preparation and fracture strength testing

Mandibular first premolars were selected for this study as these teeth were vulnerable to oblique root fracture because of wedge-shaped defect and following endodontic treatment [21]. The teeth were collected for orthodontic reasons from young adults who lived in the same area, and had very similar root forms and dimensions, as shown in Table 1. Gross destruction of coronal tooth structure was simulated by using standardized root-face preparations. The obliquely broken teeth may also need SCLM [16–18] or OFEM [19, 20], which facilitates placement of a long complete ferrule to potentially improve the fracture resistance of the residual roots [3, 5–8]. All the cores and ferrule preparations were machined by the same person (Q-F M) using the same milling device, to decrease the personal error at a minimum.

The roots of the restored teeth were coated with silicone rubber and embedded in acrylic resin. The moduli of elasticity of these materials approximated those of the viscoelastic periodontal ligament and the alveolar bone, respectively [22, 23]. The in vitro dynamic loading testing and the following unidirectional static loading forces were both used in this and many other studies of teeth fractures, in order to closely simulate the complex oral mastication [3, 11]. The oblique force applied at 135° from the long axis of the mandibular premolar was employed to simulate functional working-side buccal cuspid loading [24].

### Fracture resistance of restored premolars

**Fig. 4:**
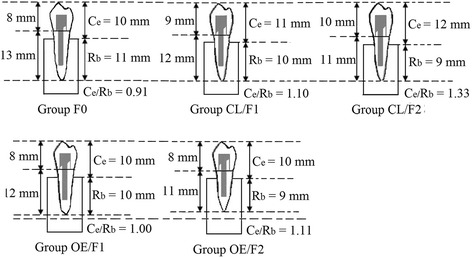
Effective crown length (Ce) to root length in bone (Rb) ratios (Ce/Rb) for restored teeth: group codes are defined in Fig. 1

The effective clinical crown length (Ce) to embedded root length (Rb) ratio of the restored mandibular premolar with its root embedded in acrylic resin, is defined as “the physical relationship between the portion of the tooth not in the alveolar bone and the portion within the alveolar bone, as determined radiographically” [25]. When the oblique force applied to the buccal cuspid of the premolar, the tooth with its root embedded in the acrylic resin could be considered as a Class І lever, with the fulcrum in the cervical portion [26]. The fracture resistance of endodontically treated teeth is dependent on the level of surrounding supporting alveolar bone and the reduction of alveolar bone height may lead to an increased risk of tooth failure [27, 28]. SCLM increases the effective clinical crown length of the lever (effort arm) during the cervical ferrule preparation, with the embedded root length (resistance arm) decreased. Thus, the Ce/Rb ratio for groups CL/F1 and CL/F2 in this study were 1.10 and 1.33 (20.9 and 46.2% greater than the control group F0), and the mean fracture resistance were 9.9 and 27.7% lower than that for the control group F0, respectively (Table 2 and Fig. 4). OFEM only decreases the embedded root length of the tooth, remaining the height of the crown portion the same as that in control group F0. However, with an increase in ferrule height prepared in the cervical portion and the reduction of root length embedded in bone, the diameter and dentine bulk of the residual root was decreased towards the root apex because of the root taper. Thus, the mean fracture resistance for group OE/F1 and OE/F2 were 5.0 and 24.8% lower than that for the control group, respectively (Table 2).

The crown-to-root ratio is one of the main variables in evaluating the suitability of a tooth as an abutment for a fixed partial denture [4, 29, 30]. Shillingburg suggested 1:1 as a minimum ratio for a prospective abutment under normal circumstances [30]. In this study, the crown-to-root ratio was defined as the effective clinical crown length (Ce) to embedded root length (Rb) ratio (Ce/Rb). As shown in Fig. 4, the Ce/Rb ratio for the control group F0 was 0.91, which was less than the minimum suggested. In contrast, the Ce/Rb ratios for groups CL/F1, CL/F2, OE/F1 and OE/F2 were 1.10, 1.33, 1.00 and 1.11 respectively, all of which were equal to, or more than, 1:1 (the acceptable minimum ratio), which may be another reason for the variable trend of fracture resistance in this study (Table 2 and Fig. 4). The relationship between the crown-to-root ratio and the fracture resistance of the endodontically treated teeth was also supported by other in vitro study [31], but further more researches being needed for in the future.

In this in vitro study, the combination of cervical treatment methods (SCLM or OFEM) and a complete ferrule preparation decreased the fracture resistance of endodontically-treated obliquely-fractured mandibular premolar. Therefore, the null hypothesis that the fracture resistance of endodontically treated residual roots would not be affected by the ferrule length, was rejected; however, that the fracture resistance would not be affected by the cervical treatment methods, was accepted.

## Conclusions

Within the limitations of this in vitro study, the following conclusions were drawn:

1. The fracture resistance of endodontically treated premolar with a lingual-to-buccal oblique fracture and an incomplete ferrule preparation were highest.
2. The combination of cervical treatment methods (SCLM or OFEM) and a complete ferrule preparation decreased the fracture resistance of endodontically-treated mandibular premolar.

## Abbreviations

Ce/Rb: The effective clinical crown length (Ce) to embedded root length (Rb) ratio
CEJ: Cemento-enamel junction
kN: Kilonewton
OFEM: Orthodontic forced eruption method
SCLM: Surgical crown lengthening method
